# Mild-to-Moderate Croup Presentations in Patients With COVID-19 Infection

**DOI:** 10.7759/cureus.27893

**Published:** 2022-08-11

**Authors:** Danica Mathew, Jose Cucalon Calderon

**Affiliations:** 1 Pediatrics, University of Nevada Reno School of Medicine, Reno, USA

**Keywords:** viral illness, infectious disease, cardiovascular, respiratory, covid-19, croup

## Abstract

This case series will describe two pediatric patients, aged nine months and two years, who presented to the emergency department with symptoms of croup. However, they were later found to have coronavirus disease 2019 (COVID-19). The two patients had no pre-existing medical conditions, and they were treated with steroids and nebulized epinephrine as required. They were discharged home with instructions to follow up with their primary care provider and to follow isolation guidelines. This report will illustrate how upper respiratory symptoms, including those consistent with croup, can be associated with or caused by an existing COVID-19 infection. More research is needed to determine if this association can lead to more long-term complications of COVID-19. Additionally, testing for COVID-19 should be incorporated into the assessment and management of this presentation. By recognizing this, clinicians can identify the cause of the illness sooner and initiate targeted treatment more effectively.

## Introduction

Coronavirus disease 2019 (COVID-19), caused by severe acute respiratory syndrome coronavirus 2 (SARS-CoV-2), most commonly presents with fever and upper respiratory symptoms, including cough and shortness of breath. It can also be associated with nausea, vomiting, and abdominal pain, among other symptoms [1-3]. COVID-19 vaccination, when age-appropriate, continues to be the most important preventative measure to protect children from this virus [4,5-7]. Some of the factors that place children at a higher risk for severe infection are age of less than one year, prematurity, neurodevelopmental disorders (cerebral palsy or trisomy 21), obesity, and asthma [5]. However, most cases of COVID-19 in children are mild and usually managed with supportive care.

Croup, or laryngotracheitis, is a respiratory illness that is typically caused by viral infections, most commonly the parainfluenza virus. It initially presents during the winter months and manifests upper respiratory tract infections, followed by barking cough, stridor, and dyspnea. This is caused by the narrowing of the subglottic airway. Factors that may predispose pediatric patients to croup are congenital anatomic narrowing of the upper airway, history of atopy, asthma, gastroesophageal reflux disease, respiratory tract papillomas from human papillomavirus (HPV), and upper airway hemangiomas [2,3,7-9]. The severity of the presentation is measured by the Westley croup score, which evaluates multiple aspects of the presentation such as level of consciousness, cyanosis, stridor, air entry, and retractions. The management of croup is dependent on the severity of the illness [2-4,10,11]. The initial treatment involves supportive care and oxygen supplementation, along with conservative measures to keep the child comfortable [10]. Mild-moderate croup is treated with oral steroids and observation. In a severe presentation, racemic epinephrine (every 15 minutes as needed) and systemic steroids (most commonly dexamethasone or prednisone) are administered. In cases with severe symptoms, patients may require invasive mechanical ventilation [3,12].

We present a case series of two patients evaluated in the emergency department between January and February of 2022, during the Omicron wave of COVID-19. They were diagnosed with clinical croup and tested positive for COVID-19 as well.

## Case presentation

Case 1

A two-year-old female with no pertinent past medical history presented to the clinic with congestion, shortness of breath, wheezing, and a fever of 104 °F at home. She additionally had rhinorrhea and a hoarse voice. The patient’s mother denied vomiting, diarrhea, or loss of appetite. The patient’s vaccination status was not documented. She had no relevant past medical history. This patient was normally developed and had no pertinent birth history. On physical exam, she was afebrile at 97.1 °F; her oxygen saturation was 99%, respiratory rate was 20, and her blood pressure was 110/70 mmHg. Lung auscultation revealed stridor at rest, a hoarse voice, and intermittently increased work of breathing. Cardiac auscultation was normal with no murmurs appreciated; capillary refill time was also normal. The patient had no mental status changes and was alert. Based on the patient’s stridor and associated symptoms, she met the criteria for mild croup. She was prescribed a two-day course of oral prednisolone (0.15 mg/kg/day) for the treatment of croup. Her symptoms improved after treatment. She was swabbed for COVID-19 polymerase chain reaction (PCR) and influenza A/B, with nasopharyngeal sample testing positive by PCR. The parent was encouraged to use cold air and steam treatment to help alleviate stridor. The patient was observed for four hours to assess for rebound symptoms and was discharged home in stable condition. When this patient was seen by her primary care provider two weeks later, her symptoms had resolved.

Case 2

A nine-month-old male with no significant past medical history presented to urgent care for a six-day history of fever (maximum temperature of 103 °F), cough, congestion, and vomiting. The patient was initially taken to an urgent care facility, where he was discharged with supportive care. Later on the same day, he was taken to the emergency department due to the non-improvement of symptoms. His parents had recently tested positive for COVID-19 and presented with similar upper respiratory tract infection symptoms. His vaccinations were up to date for his age. A respiratory panel testing for the respiratory syncytial virus (RSV), influenza A/B, and COVID-19 was collected, and they returned negative for RSV and Influenza A/B but positive for COVID-19. The patient had no chronic health conditions and no relevant birth or developmental history. He was not taking any medications. His vital signs were as follows: temperature of 103.6 °F, pulse rate of 168, respiratory rate of 24, blood pressure of 100/60 mmHg, and oxygen saturation of 92% on room air. On physical exam, lung auscultation showed tachypnea, diffuse rhonchi, retractions, and diffuse inspiratory and expiratory wheezing. Cardiac auscultation was normal and no murmurs were appreciated. The patient was alert, and no mental status changes were noted. Chest X-ray showed perihilar and bronchial wall thickening consistent with viral bronchiolitis. The patient's clinical picture was consistent with moderate croup severity based on his chest wall retractions, decreased air entry, and the presence of stridor when agitated. He was started on racemic epinephrine (0.05 mL/kg of a 2.25% solution diluted to 3 mL) and was administered a dose of oral Decadron (0.3 mg/kg). He received two doses of racemic epinephrine during this course. The patient was observed for four hours after the second dose of racemic epinephrine to assess for additional symptoms. He was discharged home in stable condition with instructions to follow up with his primary care provider within the week. When the patient was seen by his primary care provider one week later, his symptoms had improved.

## Discussion

Croup can lead to severe outcomes in children. Pediatric patients with moderate to severe presentations are at increased risk for respiratory failure, pneumomediastinum, and pulmonary edema [13,14]. Other complications include secondary bacterial pneumonia and bacterial tracheitis. These complications are more common in patients who require intubation, which correlates with worse outcomes [15,16]. Children with recurrent episodes of croup may need evaluation from an otolaryngologist with laryngobronchoscopy to assess for underlying anatomic airway abnormalities [17-19]. It is important to recognize that the cases described in this report did not have severe presentations or poor clinical outcomes as mentioned above.

Of note, our two patients presented in 2022 with upper respiratory tract symptoms during the Omicron variant wave of COVID-19. Although these two cases both occurred epidemiologically during the Omicron variant wave of COVID-19, this is a time-based correlation, and there is no evidence to support that these symptoms were caused by the Omicron variant. Current evidence shows that there was an increase in clinical croup diagnoses at the height of the Omicron variant wave [20]. At the time of writing this report, our literature search did not yield any evidence pointing toward other COVID-19 variants associated with croup. Further evidence is needed to determine if there is an association between clinical croup and other COVID-19 variants. Both cases were found to have mild to moderate croup while testing positive for COVID-19 infection. Another possibility in these case presentations is a respiratory viral co-infection presenting as clinical croup. The patients in this report were only tested for influenza and not other known causes of croup, making that a limitation of this case series.

Currently, there is scarce data on more severe outcomes in croup-related COVID-19 [20]. However, most of the available data is consistent with typical croup presentations and outcomes, similar to the cases described in our series. We encourage other researchers to take our findings as further evidence that pediatric patients with symptoms consistent with croup should be tested for COVID 19, as this will have documented contact precaution implications supporting the current American Academy of Pediatrics (AAP) guidelines [21].

## Conclusions

New COVID-19 presentations are anticipated to be more common as the pandemic continues. This case series can help to recognize the association of COVID-19 with croup. Further investigations and examinations are needed to understand the pathophysiology of COVID-19, its relationship to croup, and how the correlation of these two infections can contribute to routine testing for COVID-19 in children.
